# Left atrial appendage occlusion: trends in demographics and in-hospital outcomes—a German nationwide analysis

**DOI:** 10.1007/s00392-024-02586-0

**Published:** 2025-01-07

**Authors:** Jakob Christoph Voran, Hatim Seoudy, Marius Leye, Benedikt Kolbrink, Kevin Schulte, Astrid Dempfle, Derk Frank, Felix Kreidel

**Affiliations:** 1 Department of Internal Medicine III, Cardiology and Critical Care, University Hospital Schleswig-Holstein, Campus Kiel, Arnold-Heller-Str. 3, 24105 Kiel, Germany; 2 DZHK (German Centre for Cardiovascular Research), Partner Site Hamburg/Kiel/Lübeck, Kiel, Germany; 3 Department of Nephrology and Hypertension, University Hospital Schleswig-Holstein, Campus Kiel, Kiel, Germany; 4 Institute of Medical Informatics and Statistics, Kiel University, University Hospital Schleswig-Holstein, Kiel, Germany; 5 Department of Cardiology, University Hospital Heidelberg, Heidelberg, Germany

**Keywords:** Left atrial appendage occlusion, Age, Germany, In-hospital mortality

## Abstract

**Background:**

LAAO is an interventional, prophylactic treatment to prevent cardioembolic stroke in patients with non-valvular atrial fibrillation.

**Aims:**

The aim of this study was to assess gender differences and age-related in-hospital course of all patients undergoing left atrial appendage occlusion (LAAO) in Germany.

**Methods:**

The Research Data Center of the Federal Statistical Office accessed interrogation of its Diagnosis Related Groups (DRG) statistics database. In a retrospective observational manner, all German in-hospital cases from 2016 to 2022 with a coded LAAO procedure were analyzed.

**Results:**

LAAO was performed on a total of 40,435 patients, 39.2% of whom were female. The relative frequency of procedures in the German male population over the age of 60 was twice as high as in the German female population. The median age was 78 (IQR: 72–82) years. Compared to 28.3% in 2016, in 2022 40.1% of all patients were over 80 years of age (increased by 152%). Cases of patients over 85 years of age increased from 7.7 to 11.4% during the same time period. We found an in-hospital death rate for patients < 70, 70–75, 80–85 and > 85 years of age of 0.8, 1.0, 1.4 and 2.2% respectively. Further, we saw significantly higher MACE rates (< 75 years: 4%, 75–85 years: 5%, > 85 years: 7%) in patients with a higher age. Gender was not significantly associated with a higher rate of in-hospital mortality.

**Conclusions:**

In Germany, LAAO is increasingly performed in older patients with a strong gender imbalance. Age was independently associated with higher in-hospital MACE and mortality rates. This data provides a further basis to balance risks and benefits of LAAO as a preventive procedure and highlights the need for further prospective studies.

**Graphical abstract:**

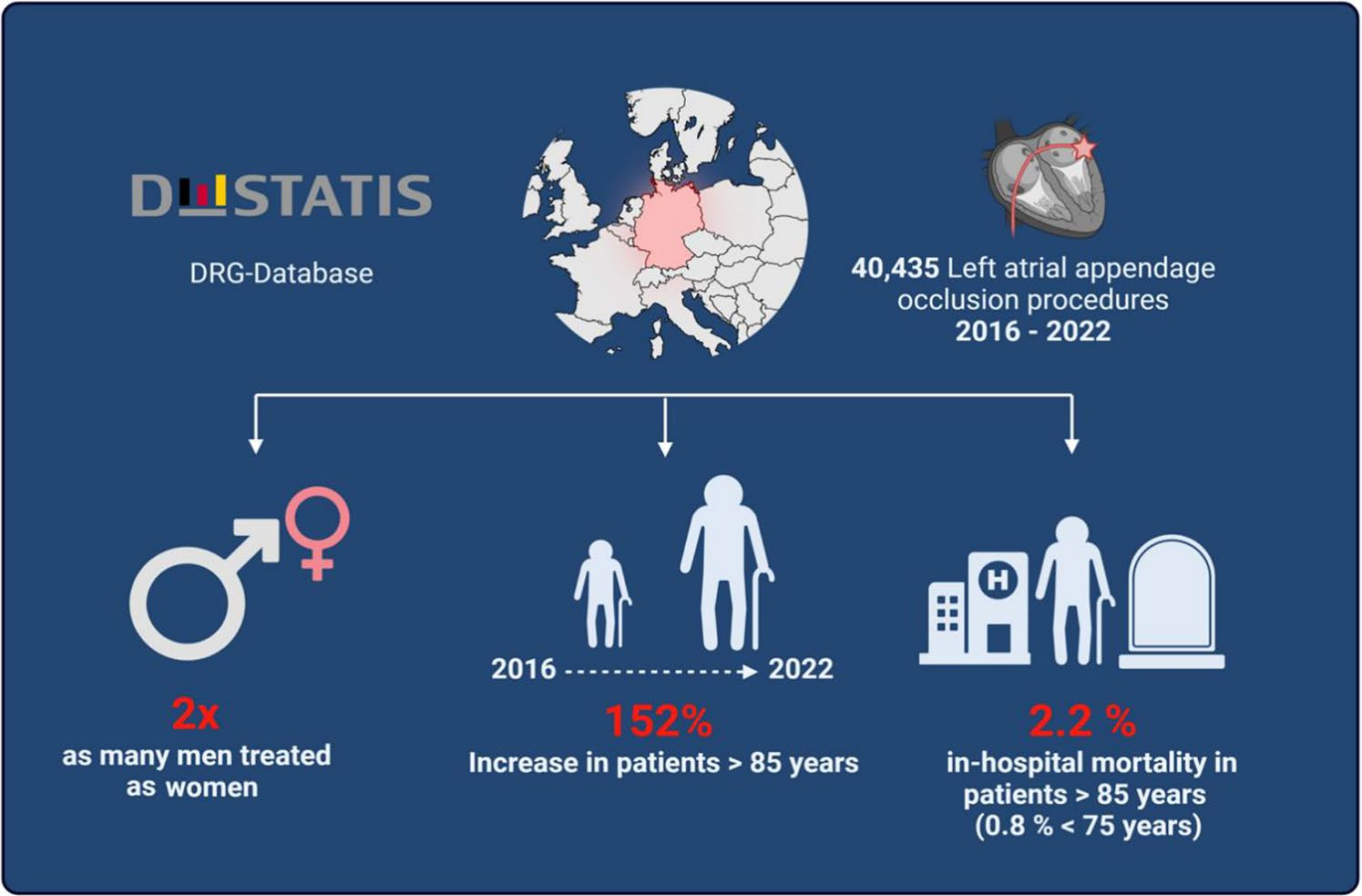

Trends in gender, demographics and in-hospital outcomes in a German nationwide analysis on all patients with an LAAO procedure in the years from 2016 to 2022.

**Supplementary Information:**

The online version contains supplementary material available at 10.1007/s00392-024-02586-0.

## Introduction

In patients with atrial fibrillation, percutaneous left atrial appendage occlusion (LAAO) has evolved as an alternative to oral anticoagulation (OAC) for stroke prevention [1]. Positive evidence supporting the catheter-based therapy stems from a small number of randomized controlled trials that included patients eligible also for OAC [2–4].

In clinical practice oftentimes LAAO is performed in older patients who are ineligible for OAC due to high-risk or bleeding complications [5]. So far, strong evidence on the efficacy and safety of LAAO in higher-risk populations is lacking as randomized-controlled investigations are still pending (CLOSURE AF; ClinicalTrials.gov Identifier: NCT03463317). Accordingly, the guidelines of the European Society of Cardiology see a Class IIb indication (Level B) only for LAAO in patients with contraindications for long-term anticoagulant therapy [6].

With aging populations and a high prevalence of atrial fibrillation LAAO is believed to address the clinical need of stroke prevention in high-risk patients who cannot tolerate OAC. On the other hand, LAAO comes with inherit risks for periprocedural complications that may increase with patients’ age and comorbidities and potentially neutralize the benefits of reducing stroke risk of thromboembolic etiology especially in elderly or high-risk patients [7].

Uncertainties regarding indication and associated benefits especially apply to female patients. A meta-analysis including 111,775 patients who underwent LAAO found that women were significantly underrepresented in clinical trials (enrollment approx. 30% only) and were more likely to have periprocedural complications [8].

As there is no prospective nationwide registry worldwide for LAAO (neither EU nor US), data on recent indications and performance trends from large samples are sparse. German authorities provide scientific access to a nationwide Diagnosis Related Groups (DRG) statistic database that includes information on in-hospital events [9, 10]. With growing appraisal of the challenges of LAAO [11], we sought to investigate the most recent trends in LAAO in Germany with special regard to gender, demographics and in-hospital mortality in relation to patient age.

## Methods

For this retrospective observational study, we performed a database query of a nationwide DRG statistics database of the Research Data Center of the Federal Statistical Office. This survey includes an annual full survey of all inpatient hospital cases. The datasets contain hospital and demographic data as well as information on case history relevant to DRG-coding according to the International Classification of Diseases 10th Revision German Modification (ICD-10-GM) and the German Operation and Procedure Classification System (OPS) per individual case. To access the data we used controlled remote data processing as described before [12, 13].

To identify all cases with an LAAO procedure, we filtered the available cases with the OPS-Code 8–837.s0 (“Implantation of a permanent embolic protection system” which is a sub-code of 8–827.s “Measures for embolism protection at the left heart ear”). This OPS-Code was introduced with the OPS 2016 update. All codes used in this study are available in the supplementary Table 1. Elixhauser comorbidity score (ESS) and CHA_2_DS_2_-VASc were defined as published before [10, 14]. We defined major adverse cardiac events (MACE) as a combined endpoint of one or more events of the following group: in-hospital death, myocardial infarction, cardiac surgery, cardiopulmonary resuscitation.

The data on age and gender distribution in the total German population was taken from the Genesis database of the Federal Statistical Office and Statistical Offices of the Federal States [15]. To calculate the incidence of LAAO procedures per age group we divided the number of procedures per age-group and sex by the number of persons in the corresponding age group of the total German population.

We used R version 4.3.2 for statistical analysis. Continuous variables are described as median and interquartile range (IQR). Categorical variables are described as absolute and relative frequencies. A two-sided *p* value of < 0.05 was set as a level of significance. Trends over time were evaluated by linear regression analysis and presented as beta (β) coefficients and 95% confidence intervals (CIs). We further analyzed the impact of clinical and demographic parameters on mortality in univariate/multivariate logistic regression and calculated odds ratios (ORs) and the corresponding 95% confidence intervals (CI) using the glm function of base R.

Approval of this study was given directly by the Research Data Centre of the Federal Statistical Office of Germany (65,189 Wiesbaden, Gustav-Stresemann-Ring 11, forschungsdatenzentrum@destatis.de).

## Results

**Fig. 1:**
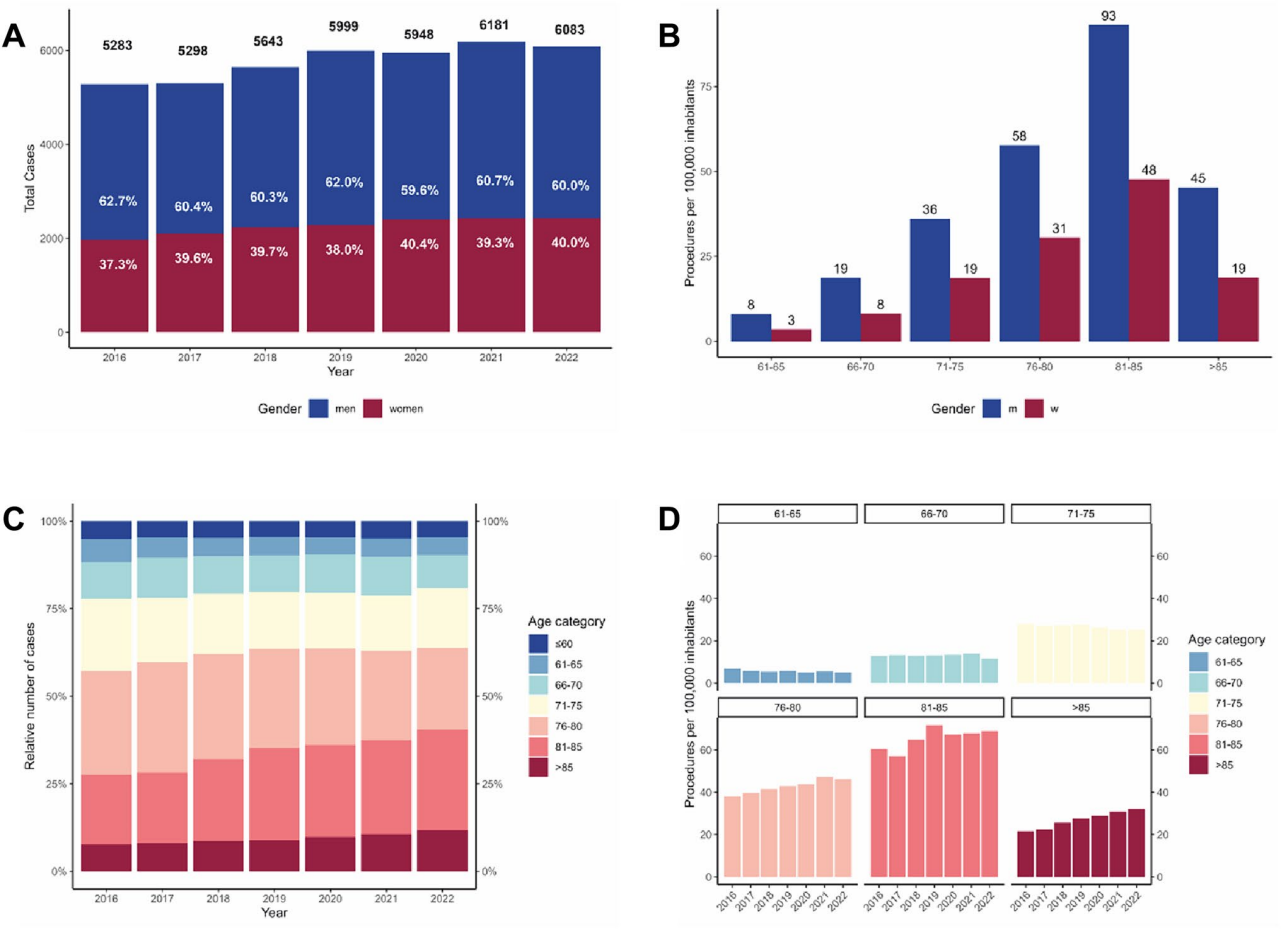
**A** Number of cases with interventional left atrial occlusion procedures by year. White text labels indicate the gender proportion per year. **B** Procedures per 100,000 inhabitants of the corresponding age population by sex **C** Proportion of cases per age category. **D** Evolution of the age-specific procedure rate per 100,000 inhabitants from 2016 to 2022

For the 7 years from 2016 to 2022, the German nationwide sample comprised 40,435 hospitalized patients who underwent a LAAO procedure. With 5283 cases in 2016 to 6083 cases in 2022 the number of yearly implantations has risen by 15%. 2021 was the year with the highest number of LAAO-procedures (*n* = 6181) during the studied time period (Fig. 1A).

### Gender

**Fig. 2:**
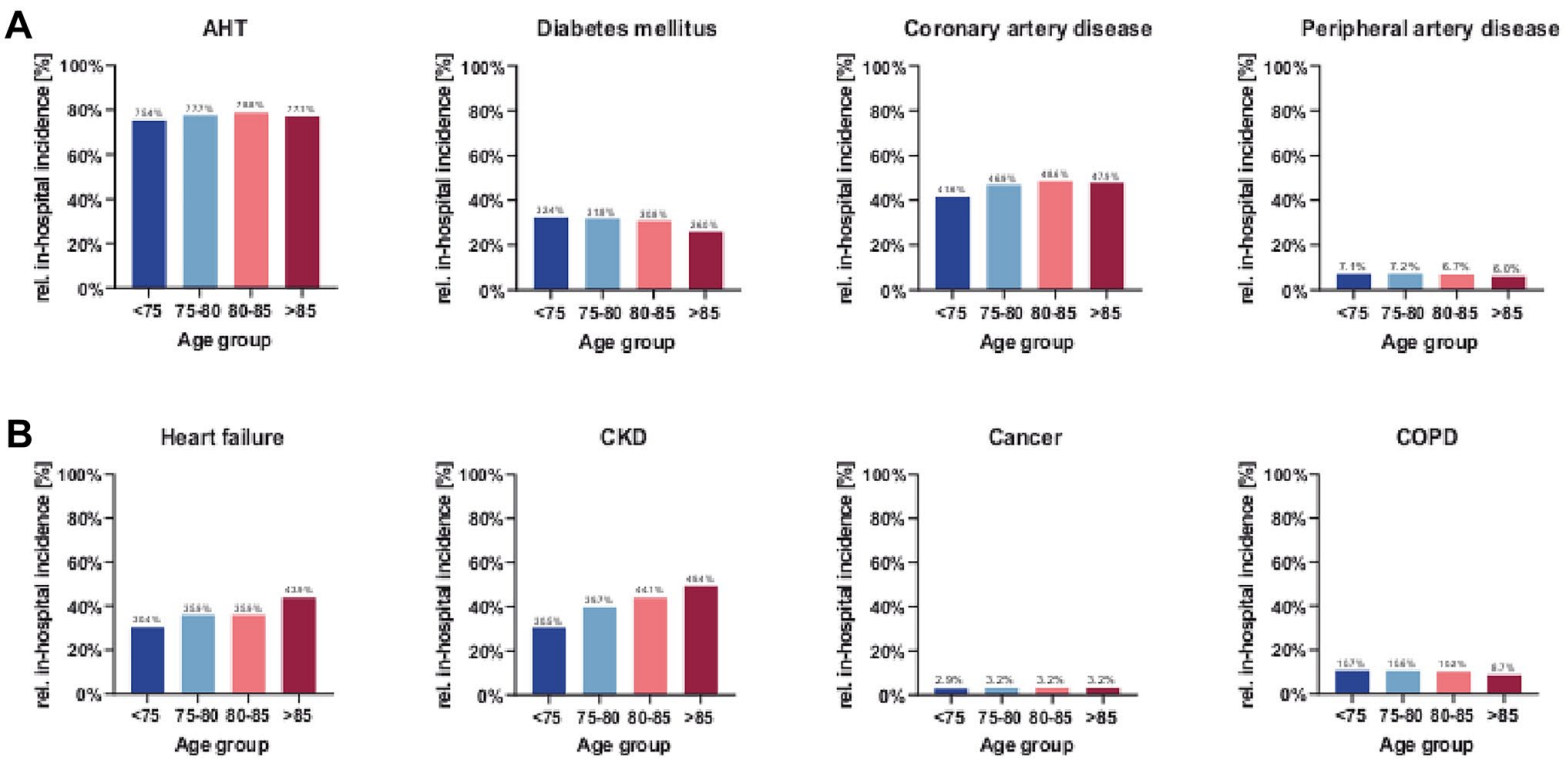
**A** Rates of cardiac risk factors per age category, **B** rates of selected comorbidities per age category. *AHT* arterial hypertension, *CKD* chronic kidney disease, *COPD* chronic obstructive pulmonary disease

An analysis of the gender distribution showed that 39.2% of the patients treated were female. This proportion remained stable over the investigated time (Fig. 1A). In the age groups of men over 75 years of age, the incidence of LAAO procedures was more than twice as high as in the corresponding female population (male vs. female procedures per 100,000 persons; 61–65 years: 8.0 vs 3.5; 66–70 years: 18.7 vs 8.1; 71–75 years: 36.1 vs 18.6; 76–80 years: 57.7 vs 30.5; 81–85 years: 93.1 vs. 47.7; > 85 years: 45.3 vs 18.7; in inhabitants over > 61 years of age the procedure rate was 1.9-fold higher in men than in women, Fig. 2B).

### Age

When analyzing the age distribution of patients undergoing treatment, we found a shift towards an older patient collective (Fig. 1C). The median age 2016 was 77 years (IQR: 72–81) and increased by two years to a median of 79 years in 2022 (IQR: 72–83). While 407 (7.7% of patients treated that year) patients over 85 years of age were treated with an LAAO in 2016, this number rose to 714 (11.7%; *β* = 0.6%/year, CI 0.5–0.8%, *p* < 0.001) patients in 2022, representing a relative increase by 152.3%. There was also an increase in the 81–85 age group from 1047 patients treated in 2016 (19.8% of patients treated that year) to 1750 patients in 2022 (28.8%; *β* = 1.5%/year, CI = 1.0–2.0%, *p* < 0.001).

This rise in procedures in the higher age groups was stronger than the natural increase associated with the ageing population (Fig. 1D). In the German population over 85 years of age, the procedures per 100,000 inhabitants rose from 21.6 in 2016 to 32.2 (*β* = 1.86/year, CI 1.6–2.1%, *p* < 0.001).

### Comorbidities

Patient characteristics, comorbidities and outcome parameters are summarized in Table 1. In 98.1% of the cases, we found an ICD-Code for atrial fibrillation, which was divided into 32.1% for permanent, 26% for persistent, and 40.8% for paroxysmal atrial fibrillation. The median CHA_2_DS_2_-VASc was 4 (IQR: 3–5).

**Table 1:** Clinical and outcome data according to age category

	Full cohort	Age category (years)			
		< 75	75–80	81–85	> 85
*n* (%)	40,435	15,364 (38.0)	11,269 (27.9)	9981 (24.7)	3821 (9.4)
Age, y (median, IQR)	78 (72–82)	70 (65–73)	78 (77–78)	83 (82–84)	87 (86–89)
Female, *n* (%)	13,421 (39.1%)	5237 (34.1)	4521 (40.1)	4312 (43.2)	1784 (46.7)
Comorbidities					
ESS, median (IQR)	4 (3–6)	4 (3–6)	4 (3–6)	4 (3–6)	5 (3–6)
CHA_2_DS_2_VASc-Score, median	4	3	4	5	5
Atrial fibrillation total	39,670 (98.1)	14,883 (96.9)	11,141 (98.9)	9882 (99.0)	3764 (98.5)
Paroxysmal	16,504 (40.8)	6737 (43.8)	4550 (40.4)	3828 (38.4)	1389 (36.4)
Persistent	10,518 (26.0)	4318 (28.1)	2998 (26.6)	2371 (23.8)	831 (21.7)
Permanent	12,993 (32.1)	3890 (25.3)	3710 (32.9)	3815 (38.2)	1578 (41.3)
Congestive heart failure	14,346 (35.5)	4675 (30.4)	4046 (35.9)	3946 (39.5)	1679 (43.9)
Coronary artery disease	18,364 (45.4)	6399 (41.6)	5282 (46.9)	4851 (48.6)	1832 (47.9)
Peripheral artery disease	2800 (6.9)	1085 (7.1)	815 (7.2)	672 (6.7)	228 (6.0)
Hypertension	31,160 (77.1)	11,588 (75.4)	8756 (77.7)	7870 (78.8)	2946 (77.1)
Diabetes mellitus	12,617 (31.2)	4973 (32.4)	3578 (31.8)	3073 (30.8)	993 (26.0)
Dyslipidemia	14,767 (36.5)	5580 (36.3)	4258 (37.8)	3641 (36.5)	1288 (33.7)
Chronic lung disease	4190 (10.4)	1644 (10.7)	1197 (10.6)	1015 (10.2)	334 (8.7)
Chronic kidney disease	15,454 (38.2)	4682 (30.5)	4479 (39.7)	4406 (44.1)	1887 (49.4)
Cancer	1244 (3.1)	441 (2.9)	365 (3.2)	316 (3.2)	122 (3.2)
In-hospital course					
Length of stay. d (median. IQR)	4 (2–8)	3 (2–6)	4 (2–8)	4 (2–9)	5 (3–11)
RBC transfusion	6147 (15.2)	1726 (11.2)	1708 (15.2)	1814 (18.2)	899 (23.5)
Pericardial effusion	354 (0.9)	102 (0.7)	106 (0.9)	88 (0.9)	58 (1.5)
Pericardiocentesis	86 (0.2)	23 (0.1)	24 (0.2)	24 (0.2)	15 (0.4)
Cardiac surgery	793 (2.0)	258 (1.7)	221 (2.0)	219 (2.2)	95 (2.5)
Acute kidney injury	2385 (5.9)	693 (4.5)	672 (6.0)	695 (7.0)	325 (8.5)
ICU	3300 (8.2)	1216 (7.9)	910 (8.1)	843 (8.4)	331 (8.7)
Outcomes					
Combined adverse events including death (MACE)	2068 (5.12)	644 (4.2)	552 (4.9)	589 (5.9)	284 (7.4)
In-hospital mortality total	456 (1.1)	121 (0.8)	111 (1.0)	141 (1.4)	83 (2.2)

In patients with a higher age, we found a higher rate of permanent atrial fibrillation (< 76 years: 25.3%; 76–80 years: 32.9%; 81–85 years: 38.2%, > 85 years: 41.3%). Measured by the ESS, we found a one-point higher mean summary score in the patients aged 85 years or older. We found no difference in the other age categories. In older patients, we found higher frequencies of chronic kidney diseases and congestive heart failure, similar frequencies of cancer diagnoses, hypertension and coronary artery disease, and lower frequencies of carotid disease, hyperlipidemia, COPD and diabetes (Fig. 2).

### Outcome

**Fig. 3:**
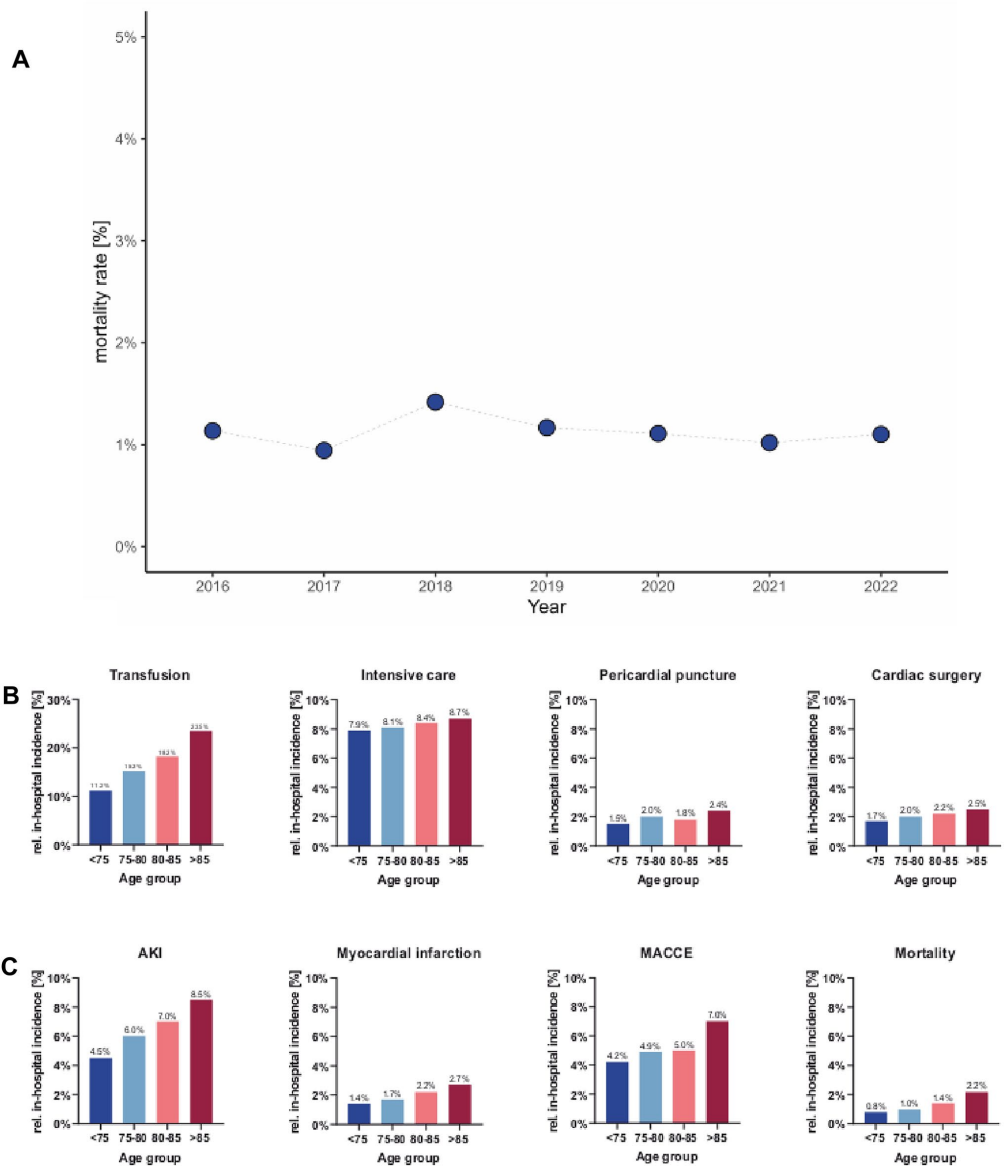
**A** In-hospital mortality rate in the years 2016 to 2022, **B** Relative in-hospital occurrence of procedures per age category, **C** Relative in-hospital incidence of complications per age category. AKI = Acute kidney injury, MACE was defined as a composite endpoint of in-hospital death, myocardial infarction, cardiac surgery and cardiopulmonary resuscitation

The overall in-hospital mortality rate was 1.13% and ranged from 0.94 to 1.42% over time with a trend towards a lower mortality in recent years (Fig. 3). We compared outcome parameters related to patient’s age categories. In older age groups we found significantly higher in-hospital death rates (Table 1). In patients under 76 years of age, 0.8% deceased during the hospital stay, in contrast, the mortality rate for patients over 85 years of age was 2.2%. In line with this, the rate of ICU treatment was the highest in patients older than 85 years. Also, we found higher rates of open heart surgery (< 76 years: 1.7%; > 85 years: 2.5%), transfusion (< 76 years: 11.2%; > 85 years: 23.5%), and pericardial effusion (< 76 years: 0.7%; > 85 years: 1.5%). In line with these findings also MACE rates were found to increase with higher age (< 75 years: 4%, 75–85 years: 5%, > 85 years: 7%). In an univariate and multivariate model age was independently associated with in-hospital death (Table 2).

**Table 2:** Univariate and multivariate analysis of baseline characteristics and comorbidities on in-hospital mortality

	Univariate			Multivariate	
	OR (95% CI)	*p* value	*q* value	OR (95% CI)	*p* value
Age (yrs)	1.05 (1.04, 1.07)	< 0.001	< 0.001	1.05 (1.03, 1.06)	< 0.001
Sex: female	1.13 (0.94, 1.26)	0.203	0.248	1.09 (0.9, 1.32)	0.4
Comorbidities					
Coronary artery disease	1.27 (1.06, 1.53)	0.011	0.019	1.08 (0.89, 1.32)	0.4
Peripheral arterial disease	1.82 (1.35, 2.40)	< 0.001	< 0.001	1.64 (1.21, 2.18)	0.001
COPD	1.55 (1.19, 1.99)	0.002	0.004	1.43 (1.09, 1.85)	0.007
Chronic kidney disease	2.05 (1.70, 2.47)	< 0.001	< 0.001	1.75 (1.44, 2.12)	< 0.001
Diabetes mellitus	1.24 (1.02, 1.50)	0.029	0.037	1.14 (0.93, 1,39)	0.2
Arterial hypertension	0.77 (0.63, 0.95)	0.014	0.020	0.69 (0.56, 0.86)	< 0.001
Prev. Card. Surgery	1.09 (0.83, 1.42)	0.522	0.522	0.89 (0.67, 1.18)	0.4

*CI* confidence interval, *COPD* chronic obstructive pulmonary disease, *OR* odds ratio

## Discussion

Here, we present a German nationwide real-world analysis of 40,435 hospital cases in which an LAAO procedure was performed. Three main findings stand out: (1) The incidence of LAAO procedures in men is more than twice as high as in women, (2) there is a relevant shift towards an older patient collective, and (3) a higher age is associated with a longer hospital stay, more severe complications and a higher in-hospital mortality.

The database used in this study had been investigated two times before with respect to LAAO procedures: Hobohm et al. examined all cases in which the previous OPS-Code for LAAO procedures was used (2011–2015; 15,895 patients) [9]; Maier et al. reported data from 2016 to 2020 (28,039 patients) and focused on the difference between endocardial occlusion and epicardial loop stitch [10]. Our study covers an additional 12,000 patients and an integration with data of the general German population, both of which allowed for a more in-depth analysis of gender and age trends and their impact on intrahospital outcome in patients undergoing LAAO.

### Differences in gender-specific incidence

Regarding gender distribution, our analysis showed a clear surplus of treated men compared to women in all age groups. This difference seems least pronounced in the highest age group (46.7% female) but in relation to the gender distribution of the total German population, the overall incidence of LAAO in women calculates to be only half as high as in men of the same age. This is a striking finding, given the reported lifetime risk of atrial fibrillation being just slightly higher in men [16] and given the evidence that women are found to potentially have more bleeding complications under OAC [17]. On the other hand, it is in line with data from the US, where in a large nationwide cohort the proportion of women was also significantly lower than the gender distribution would imply [7]. A surplus of men is a well-known phenomenon in interventional cardiology and applies to several indications [18]. We see the same and well-described biases in charge also for the field of LAAO as also in the initial randomized-controlled trials women were underrepresented [4]. The fundamental question of whether interventional techniques initially applied mainly in male patients can be transferred and equally applied in women is still open. For LAAO there is a signal from a meta-analysis of 16 studies (111,775 patients) that the periprocedural complication rate is higher in women [8]. In our analysis, female gender was also associated with a higher risk of in-hospital death, but this was not statistically significant.

### Shift towards an older patient collective

Our finding of a clear shift towards an ever-aging patient population was not discussed in previous publications, but the analysis of Hobohm et al. had already shown an increasing mean age over the investigated time period. From 2011 to 2022 the median age rose from 75 (IQR: 70–79) to 79 (IQR: 72–83) years. While patients in the 75–80 age group still accounted for the majority of patients treated in 2016, patients in the 80–85 age group made up the majority in 2022. Two US-American nationwide sample studies on patients undergoing LAAO have been published [7, 19]. Median age is comparable to our population. Both do not report age trends for the much shorter time periods analyzed. Freeman et al., however, report similar age categories with the group with the highest age already comprising a high number of patients (14.1%) in the years from 2016 to 2018.

### Periprocedural safety

In line with the two previous nationwide German analyses, we also found no significant improvement in procedural safety over the investigated years (in-hospital mortality ranged from 0.9 to 1.4% from 2013 to 2020 [10]). A recently published analysis from a prospective multicenter German registry [20], including 638 patients from 38 hospitals between 2014 and 2016, reported significantly fewer MACE events (0.6%) and fewer combined major complications (4.4%). It should be noted here that the patient collective was on average 4 years younger than in the total German cohort reported here. Compared to the US NCDR’s LAAO Registry 2016–2018, German patients were slightly younger and had a higher percentage of permanent atrial fibrillation and a higher incidence of severe comorbidities like chronic kidney disease (38.2% vs 13.6%). Notably, the in-hospital mortality was more than fivefold higher (1.1% vs. 0.19%) in the German cohort. This is a striking finding, also as in-hospital mortality in the other US-American nationwide study was similarly low at 0.14% [19]. Here the analyzed time period and patient age again were very comparable to the NCDR’s Registry, differentiated information on comorbidities was sparse though. Further real-world data from the US also took readmissions within 90 days into account and found a mortality rate of 0.53% [21]. Even though this is more than double the rate of the reported US in-hospital mortality in the nationwide samples, it still is lower than in Germany. Most recent evidence from an all-comers registry, the Amulet registry (Europe, Australia, Israel, Chile, Hong Kong), showed 3 procedure-related deaths (0.28%), further mortality rates were 0.59% within 30 days 8.4% within the first year [22]. In all four randomized controlled trials analyzing LAAO only one procedure-related death (0.5%) was reported in the PRAGUE-17 study, in all others there was none [2–4, 23].

The differences in reported mortality rates between Germany and the US nationwide cohorts are difficult to understand. Due to the nature of retrospective cohort studies more granular data on timing and etiology of mortality is not available. In general, interventional technique and periprocedural care should by all means be comparable in the US and Europe. Nonetheless, we also noted higher rates of severe complications that may potentially have led to more intra-hospital deaths (i.e., the rate of cardiac surgery in our population was 2.0% compared to 0.24% in the NCDR’s LAAO Registry). Further, we consider two differences in patient management that may explain the different outcomes. One, as eluded to before, the patients we report appear to be significantly sicker. Secondly, it may be for a different timing of indication and intervention. The more LAAO is undergone as an elective procedure the less comorbidities should play into role. Irrespectively, a reporting bias cannot be ruled out, we believe though that mortality as the most definite outcome should be explicitly well coded in Germany and the US.

How relevant patient characteristics are for the occurrence of MACE is shown by our analysis as rates of in-hospital mortality, pericardial effusion, acute kidney injury and even heart surgery are highest in the group with the highest patient age (in-hospital mortality 2.2%, MACE 7.4%). Balancing the risks and benefits of an interventional procedure is crucial especially in a prophylactic treatment like LAAO. With increasing age, the risk/benefit ratio seems to be negatively impacted as already in-hospital MACE rates are higher than predicted annular stroke rates (see CHA_2_DS_2_VASc-Score) [7]. Notably, also transfusion rates were highest in the oldest age group. It is unclear whether these were triggered by bleeding complications or reflect the presumed indication leading to LAAO—a relevant bleeding event under OAC.

Definitely, these data underscore the need for prospective randomized trials addressing patient populations that differ from the initially investigated one by age and also by gender to provide further evidence for the pros and cons of interventional stroke prevention in patients with atrial fibrillation.

### Limitations

This study is based on a DRG registry containing ICD and OPS discharge codes coded primarily for reimbursement. This could lead to under-and overreporting of well-reimbursed procedures and diseases. The endpoints presented here were selected against this background; we are convinced that reporting biases are equally distributed in the groups presented. On the other hand, we present real-world data without selection bias as in RCTs and reflect the reality of care in Germany.

## Conclusions

With this nationwide cohort study, we report on the recent trends of LAAO therapy in Germany over the 7 years from 2016 to 2022. The data show a significant gender imbalance with an incidence of LAAO in men being twice as high as in women. Secondly, a further increase in the number of LAAO procedures and an accompanying increase in patient age was noted. Further, a strong association was found between patient age, comorbidities and MACE. In-hospital mortality in patients > 85 years of age was high with 2.2%. These results highlight the importance to correctly and individually weigh the risks and benefits of prophylactic LAAO, especially in elderly patients.

## Supplementary Information

Below is the link to the electronic supplementary material.

Supplementary file1 (DOCX 33 KB)

## Data Availability

All the data used in the nationwide secondary data analysis are available for researchers upon request from the DRG statistics of the Research Data Center of the Federal Statistical Office.

## Abbreviations

DRG: Diagnosis related groups
ICD: International classification of diseases
LAA: Left atrial appendage
LAAO: Left atrial appendage occlusion
OAC: Oral anticoagulation
OPS: German operation and procedure classification system
MACE: Major adverse cardiac event

## References

[1] Holmes DR, Korsholm K, Rodés-Cabau J, Saw J, Berti S, Alkhouli MA (2023) Left atrial appendage occlusion. EuroIntervention 18:e1038–e1065 36760206 10.4244/EIJ-D-22-00627 PMC9909459

[2] Osmancik P, Herman D, Neuzil P et al (2020) Left atrial appendage closure versus direct oral anticoagulants in high-risk patients with atrial fibrillation. J Am Coll Cardiol 75:3122–3135 32586585 10.1016/j.jacc.2020.04.067

[3] Holmes DRJ, Kar S, Price MJ et al (2014) Prospective randomized evaluation of the watchman left atrial appendage closure device in patients with atrial fibrillation versus long-term warfarin therapy: the PREVAIL trial. J Am Coll Cardiol 64:1–12 24998121 10.1016/j.jacc.2014.04.029

[4] Holmes DR, Reddy VY, Turi ZG et al (2009) Percutaneous closure of the left atrial appendage versus warfarin therapy for prevention of stroke in patients with atrial fibrillation: a randomised non-inferiority trial. Lancet 374:534–542 19683639 10.1016/S0140-6736(09)61343-X

[5] Labori F, Bonander C, Persson J, Svensson M (2021) Clinical follow-up of left atrial appendage occlusion in patients with atrial fibrillation ineligible of oral anticoagulation treatment-a systematic review and meta-analysis. J Interv Card Electrophysiol 61:215–225 33580847 10.1007/s10840-021-00953-9 PMC8324592

[6] Van Gelder IC, Rienstra M, Bunting KV et al (2024) 2024 ESC Guidelines for the management of atrial fibrillation developed in collaboration with the European Association for Cardio-Thoracic Surgery (EACTS). Eur Heart J 45:3314–3414 39210723 10.1093/eurheartj/ehae176

[7] Freeman JV, Varosy P, Price MJ et al (2020) The NCDR left atrial appendage occlusion registry. J Am Coll Cardiol 75:1503–1518 32238316 10.1016/j.jacc.2019.12.040 PMC7205034

[8] Mathai SV, Sohal S, Flatow E et al (2023) Sex differences in periprocedural and long-term outcomes following transcatheter left atrial appendage occlusion: a systematic review and meta-analysis. Cardiovasc Revasc Med 48:23–31 36336589 10.1016/j.carrev.2022.10.002

[9] Hobohm L, von Bardeleben RS, Ostad MA et al (2019) 5-year experience of in-hospital outcomes after percutaneous left atrial appendage closure in Germany. JACC Cardiovasc Interv 12:1044–1052 31171280 10.1016/j.jcin.2019.04.002

[10] Maier A, Kaier K, Heidt T, Westermann D, von Zur MC, Grundmann S (2023) Catheter based left atrial appendage closure in-hospital outcomes in Germany from 2016 to 2020. Clin Res Cardiol 113:1419 37698619 10.1007/s00392-023-02299-w PMC11420385

[11] Alkhouli M, De Backer O, Ellis CR et al (2023) Peridevice leak after left atrial appendage occlusion: incidence, mechanisms, clinical impact, and management. JACC Cardiovasc Interv 16:627–642 36990553 10.1016/j.jcin.2022.12.006

[12] Kuron D, Voran JC, von Samson-Himmelstjerna FA et al (2023) Epidemiology of haemophagocytic lymphohistiocytosis at the population level in Germany. Br J Haematol 201:285–289 36535304 10.1111/bjh.18617

[13] Samson-Himmelstjerna FAK, Voran JCN, Kolbrink B, Schulte K (2017) Use of immunoadsorption and plasma exchange for treating anti-glomerular basement membrane disease: clinical experience in Germany. Clin J Am Soc Nephrol 12:1162–1172 28515156 10.2215/CJN.01380217 PMC5498345

[14] Quan H, Sundararajan V, Halfon P et al (2005) Coding algorithms for defining comorbidities in ICD-9-CM and ICD-10 administrative data. Med Care 43:1130–1139 16224307 10.1097/01.mlr.0000182534.19832.83

[15] Genesis database of the Federal Statistical Office and Statistical Offices of the Federal States. Available at: https://www-genesis.destatis.de/genesis/online.

[16] Lloyd-Jones DM, Wang TJ, Leip EP et al (2004) Lifetime risk for development of atrial fibrillation: the Framingham Heart Study. Circulation 110:1042–1046 15313941 10.1161/01.CIR.0000140263.20897.42

[17] Ferroni E, Denas G, Gennaro N, Fedeli U, Pengo V (2022) Gender related differences in gastrointestinal bleeding with oral anticoagulation in atrial fibrillation. J Cardiovasc Pharmacol Ther 27:10742484211054608 34994209 10.1177/10742484211054609

[18] Liu E, Hsueh L, Kim H, Vidovich MI (2018) Global geographical variation in patient characteristics in percutaneous coronary intervention clinical trials: a systematic review and meta-analysis. Am Heart J 195:39–49 29224645 10.1016/j.ahj.2017.09.003 PMC5728179

[19] Thevathasan T, Degbeon S, Paul J et al (2023) Safety and healthcare resource utilization in patients undergoing left atrial appendage closure-a nationwide analysis. J Clin Med 12:4573 37510689 10.3390/jcm12144573 PMC10380523

[20] Ansari U, Brachmann J, Lewalter T et al (2024) LAA occlusion is effective and safe in very high-risk atrial fibrillation patients with prior stroke: results from the multicentre German LAARGE registry. Clin Res Cardiol. 10.1007/s00392-024-02376-8 38294498 10.1007/s00392-024-02376-8 PMC11420338

[21] Kogan EV, Sciria CT, Liu CF et al (2023) Early stroke and mortality after percutaneous left atrial appendage occlusion in patients with atrial fibrillation. Stroke 54:947–954 36866671 10.1161/STROKEAHA.122.041057

[22] Landmesser U, Tondo C, Camm J et al (2018) Left atrial appendage occlusion with the AMPLATZER Amulet device: one-year follow-up from the prospective global Amulet observational registry. EuroIntervention 14:e590–e597 29806820 10.4244/EIJ-D-18-00344

[23] Lakkireddy D, Thaler D, Ellis CR et al (2021) Amplatzer amulet left atrial appendage occluder versus watchman device for stroke prophylaxis (Amulet IDE): a randomized. Controlled Trial Circ 144:1543–1552 10.1161/CIRCULATIONAHA.121.057063 PMC8570346 34459659

