# Supplementary material for: Left atrial appendage occlusion: trends in demographics and in-hospital outcomes—a German nationwide analysis
Source: Clin Res Cardiol. 2025 Jan 7;115(6):927–36. doi: 10.1007/s00392-024-02586-0 (PMC13161337; doi:10.1007/s00392-024-02586-0)
Supplement: Supplementary file 1 — Supplementary file1 (DOCX 33 KB) [file 392_2024_2586_MOESM1_ESM.docx]

# Cases of interest

**ICD10-GM / OPS Description (German) ICD10-GM / OPS Description code ICD/OPS**

Perkutan-transluminale Gefäßintervention an Herz und Koronargefäßen: Maßnahmen zur Embolieprotektion am linken Herzohr: Implantation eines permanenten Embolieprotektionssystems left atrial appendage occlusion 8-837.s0 OPS

# Outcome

**ICD10-GM / OPS Description (German) ICD10-GM / OPS Description ICD/OPS Variable Code**

Akuter Myokardinfarkt myocardial infarction icd myocardial infarction I21.-

Hirninfarkt stroke icd stroke I63.-

Intrazerebrale Blutung intra cerebral bleeding icd intra cerebral bleeding I61.-

Hämoperikard, anderenorts nicht klassifiziert Hemopericardium, not elsewher icd pericardial effusion I31.2

Herzbeuteltamponade Pericardial effusion (non-inflam icd pericardial effusion I31.80

Therapeutische perkutane Punktion von Organen des Thorax: Perikard Therapeutic percutaneous punc ops pericardial puncture 8-152.0

Perikardiotomie und Kardiotomie Pericardiotomy and cardiotomy ops pericardiotomy 5-370

Intensivmedizinische Komplexbehandlung (Basisprozedur) Complex intensive care treatmen ops intensive care 8-980

Aufwendige intensivmedizinische Komplexbehandlung (Basisprozedur) Complex intensive care treatmen ops intensive care 8-98f

Transfusion von Vollblut, Erythrozytenkonzentrat und Thrombozytenkonzentrat: Erythrozytenkonzentrat: 1 TE bis unter 6 TE Transfusion of whole blood, ery ops transfusion up to 6 TE 8-800.c0

Transfusion von Vollblut, Erythrozytenkonzentrat und Thrombozytenkonzentrat: Erythrozytenkonzentrat: 6 TE bis unter 11 TE Transfusion of whole blood, red ops transfusion more than 6 TE 8-800.c1

Transfusion von Vollblut, Erythrozytenkonzentrat und Thrombozytenkonzentrat: Erythrozytenkonzentrat: 11 TE bis unter 16 TE Transfusion of whole blood, ery ops transfusion more than 6 TE 8-800.c2

Transfusion von Vollblut, Erythrozytenkonzentrat und Thrombozytenkonzentrat: Erythrozytenkonzentrat: 16 TE bis unter 24 TE Transfusion of whole blood, ery ops transfusion more than 6 TE 8-800.c3

Transfusion von Vollblut, Erythrozytenkonzentrat und Thrombozytenkonzentrat: Erythrozytenkonzentrat: 24 TE bis unter 32 TE Transfusion of whole blood, ery ops transfusion more than 6 TE 8-800.c4

Transfusion von Vollblut, Erythrozytenkonzentrat und Thrombozytenkonzentrat: Erythrozytenkonzentrat: 32 TE bis unter 40 TE Transfusion of whole blood, ery ops transfusion more than 6 TE 8-800.c5

Transfusion von Vollblut, Erythrozytenkonzentrat und Thrombozytenkonzentrat: Erythrozytenkonzentrat: 40 TE bis unter 48 TE Transfusion of whole blood, ery ops transfusion more than 6 TE 8-800.c6

Transfusion von Vollblut, Erythrozytenkonzentrat und Thrombozytenkonzentrat: Erythrozytenkonzentrat: 48 TE bis unter 56 TE Transfusion of whole blood, ery ops transfusion more than 6 TE 8-800.c7

Kardiale oder kardiopulmonale Reanimation Cardiac or cardiopulmonary resuops Cardiac or cardiopulmonary resuscitation 8-771

Akutes Nierenversagen Acute renal injury icd Acute renal injury N17

Andere Operationen am Thorax: N.n.bez. Other operations on the thorax: ops Cardiac surgery 5-35

Zusatzinformationen zu Operationen an Herzklappen: Anwendung spezieller Methoden bei minimalinvasivem Mitralklappenersatz: Sonstige Additional information on opera ops Cardiac surgery 5-36

Andere Operationen an den Koronargefäßen: N.n.bez. Other operations on the coronar ops Cardiac surgery 5-37

# Comorbidities

ICD10-GM / OPS Description (German) ICD10-GM / OPS Description Variable ICD/OPS code

Essentielle (primäre) Hypertonie aterial hypertension aterial hyp icd I10

Vorhofflimmern und Vorhofflattern Atrial fibrillation and atrial flutter Atrial fibrill icd I48

Vorhofflimmern, paroxysmal Atrial fibrillation, paroxysmal Atrial fibrill icd I48.0

Vorhofflimmern, persistierend Atrial fibrillation, persistent Atrial fibrill icd I48.1

Vorhofflimmern, permanent Atrial fibrillation, permanent Atrial fibrill icd I48.2

Reine Hypercholesterinämie Pure hypercholesterolemia hyperlipid icd E78.0

Reine Hypertriglyzeridämie Pure hypertriglyceridemia hyperlipid icd E78.1

Gemischte Hyperlipidämie Mixed hyperlipidemia hyperlipid icd E78.2

Hyperchylomikronämie Hyperchylomicronemia hyperlipid icd E78.3

Sonstige Hyperlipidämien Other hyperlipidemias hyperlipid icd E78.4

Hyperlipidämie, nicht näher bezeichnet Hyperlipidemia, unspecified hyperlipid icd E78.5

Chronische ischämische Herzkrankheit coronary artery disease coronary a icd I25

Vorhandensein eines aortokoronaren Bypasses Presence of an aortocoronary by coronary a icd Z95.1

Vorhandensein eines aortokoronaren Bypasses Presence of aortocoronary bypa previous c icd Z95.1

Vorhandensein einer künstlichen Herzklappe Presence of an artificial heart val previous c icd Z95.2

Vorhandensein einer xenogenen Herzklappe Presence of a xenogeneic heart v previous c icd Z95.3

Vorhandensein eines anderen Herzklappenersatzes Presence of other heart valve rep previous c icd Z95.4

Herzinsuffizienz congestive heart failure congestiveicd I50

Sonstige chronische obstruktive Lungenkrankheit chronic obstructive lung disease chronic ob icd J44

Chronische Nierenkrankheit chronic kidney disease chronic kidicd N18

Diabetes mellitus, Typ 1 Diabetes mellitus, type 1 diabetes icd E10

Diabetes mellitus, Typ 2 Diabetes mellitus, type 2 diabetes icd E11

Diabetes mellitus in Verbindung mit Fehl- oder Mangelernährung [Malnutrition] Diabetes mellitus in connection diabetes icd E12

Sonstiger näher bezeichneter Diabetes mellitus Other specified diabetes mellitus diabetes icd E13

Nicht näher bezeichneter Diabetes mellitus Unspecified diabetes mellitus diabetes icd E14

Bösartige Neubildungen als Primärtumoren an mehreren Lokalisationen Malignant neoplasms as primary cancer icd D0

Carcinoma in situ, nicht näher bezeichnet cacinoma in situ cancer icd D1

Sonstige und nicht näher bezeichnete Infektionskrankheiten Cancer cancer icd C

Verschluss und Stenose der A. carotis carotid disease carotid dis icd I65.2

Atherosklerose der Extremitätenarterien Atherosclerosis of the limb arteri Peripheral icd I70.2

Atherosklerose sonstiger Arterien Atherosclerosis of other arteries Peripheral icd I70.8

Generalisierte und nicht näher bezeichnete Atherosklerose Generalized and unspecified ath Peripheral icd I70.9

Periphere Gefäßkrankheit, nicht näher bezeichnet Peripheral vascular disease, uns Peripheral icd I73.9

Adipositas obesity obesity icd E66.-

Atherosklerose Atherosclerosis vascular d icd I70.-

Diabetes mellitus, Typ 1: Mit peripheren vaskulären Komplikationen Diabetes mellitus, type 1: With p vascular d icd E10.5

Diabetes mellitus, Typ 2: Mit peripheren vaskulären Komplikationen Diabetes mellitus, type 2: With p vascular d icd E11.5

Sonstiger näher bezeichneter Diabetes mellitus: Mit peripheren vaskulären Komplikationen Other specified diabetes mellitus vascular d icd E13.5

Nicht näher bezeichneter Diabetes mellitus: Mit peripheren vaskulären Komplikationen Unspecified diabetes mellitus: Wvascular d icd E14.5

Aortenaneurysma und -dissektion Aortic aneurysm and dissection vascular d icd I71

Thrombangiitis obliterans [Endangiitis von-Winiwarter-Buerger] Thrombangiitis obliterans [Enda vascular d icd I73.1

Sonstige näher bezeichnete periphere Gefäßkrankheiten Other specified peripheral vascu vascular d icd I73.8

Periphere Gefäßkrankheit, nicht näher bezeichnet Peripheral vascular disease, uns vascular d icd I73.9

Arterienstriktur Arterial stricture vascular d icd I77.1

Aortenaneurysma bei anderenorts klassifizierten Krankheiten Aortic aneurysm in diseases clas vascular d icd I79.0

Periphere Angiopathie bei anderenorts klassifizierten Krankheiten Peripheral angiopathy in disease vascular d icd I79.2

Chronische Gefäßkrankheiten des Darmes Chronic vascular diseases of the vascular d icd K55.1

Sonstige Gefäßkrankheiten des Darmes Other vascular diseases of the in vascular d icd K55.8

Gefäßkrankheit des Darmes, nicht näher bezeichnet Vascular disease of the intestine vascular d icd K55.9

Vorhandensein von sonstigen kardialen oder vaskulären Implantaten oder Transplantaten Presence of other cardiac or vascvascular d icd Z95.8

Vorhandensein von kardialem oder vaskulärem Implantat oder Transplantat, nicht näher bezeichnet Presence of cardiac or vascular i vascular d icd Z95.9

Verschluss und Stenose der A. carotis Occlusion and stenosis of the cavascular d icd I65.2

Ischämische Kardiomyopathie Ischemic cardiomyopathy vascular d icd I25.5

Akuter Myokardinfarkt Acute myocardial infarction vascular d icd I21

Rezidivierender Myokardinfarkt Recurrent myocardial infarction vascular d icd I22

Vorhandensein eines aortokoronaren Bypasses Presence of an aortocoronary by vascular d icd Z95.1

Chronische ischämische Herzkrankheit Chronic ischemic heart disease vascular d icd I25

Sonstige akute ischämische Herzkrankheit Other acute ischemic heart disea vascular d icd I24

Zerebrale transitorische Ischämie und verwandte Syndrome Cerebral transient ischemia and Stroke, TIA icd G45

Subarachnoidalblutung Subarachnoid hemorrhage Stroke, TIA icd I60

Intrazerebrale Blutung Intracerebral hemorrhage Stroke, TIA icd I61

Sonstige nichttraumatische intrakranielle Blutung Other non-traumatic intracranial Stroke, TIA icd I62

Hirninfarkt Cerebral infarction Stroke, TIA icd I63

Lungenembolie Pulmonary embolism Stroke, TIA icd I26

Embolie und Thrombose der Aorta abdominalis Embolism and thrombosis of th Stroke, TIA icd I74.01

Embolie und Thrombose der Aorta abdominalis Embolism and thrombosis of th Stroke, TIA icd I74.09

Embolie und Thrombose sonstiger und nicht näher bezeichneter Abschnitte der Aorta Embolism and thrombosis of ot Stroke, TIA icd I74.11

Embolie und Thrombose der Arterien der oberen Extremitäten Embolism and thrombosis of th Stroke, TIA icd I74.2

Embolie und Thrombose der Arterien der unteren Extremitäten Embolism and thrombosis of th Stroke, TIA icd I74.3

Embolie und Thrombose der A. iliaca Embolism and thrombosis of th Stroke, TIA icd I74.5

Embolie und Thrombose sonstiger Arterien Embolism and thrombosis of ot Stroke, TIA icd I74.8

Embolie und Thrombose nicht näher bezeichneter Arterie Embolism and thrombosis of unStroke, TIA icd I74.9

Folgen eines Schlaganfalls, nicht als Blutung oder Infarkt bezeichnet Consequences of a stroke, not d Stroke, TIA icd I694

Essentielle (primäre) Hypertonie Essential (primary) hypertension hypertens icd I10

Hypertensive Herzkrankheit Hypertensive heart disease hypertens icd I11

Hypertensive Nierenkrankheit Hypertensive kidney disease hypertens icd I12

Hypertensive Herz- und Nierenkrankheit Hypertensive heart and kidney d hypertens icd I13

Sekundäre Hypertonie Secondary hypertension hypertens icd I15

# ESS

ICD_10_GM_Beschreibung Variable ICD/OPS code

Ulcus ventriculi: Chronisch, ohne Blutung oder Perforation Peptic ulcer disease excluding bl icd K25.7

Ulcus ventriculi: Weder als akut noch als chronisch bezeichnet, ohne Blutung oder Perforation Peptic ulcer disease excluding bl icd K25.9

Ulcus duodeni: Chronisch, ohne Blutung oder Perforation Peptic ulcer disease excluding bl icd K26.7

Ulcus duodeni: Weder als akut noch als chronisch bezeichnet, ohne Blutung oder Perforation Peptic ulcer disease excluding bl icd K26.9

Ulcus pepticum, Lokalisation nicht näher bezeichnet: Chronisch, ohne Blutung oder Perforation Peptic ulcer disease excluding bl icd K27.7

Ulcus pepticum, Lokalisation nicht näher bezeichnet: Weder als akut noch als chronisch bezeichnet, ohne Blutung oder Perforation Peptic ulcer disease excluding bl icd K27.9

Ulcus pepticum jejuni: Chronisch, ohne Blutung oder Perforation Peptic ulcer disease excluding bl icd K28.7

Ulcus pepticum jejuni: Weder als akut noch als chronisch bezeichnet, ohne Blutung oder Perforation Peptic ulcer disease excluding bl icd K28.9

Infektiöse und parasitäre Krankheiten infolge HIV-Krankheit [Humane Immundefizienz-Viruskrankheit] AIDS/HIV icd B20

Bösartige Neubildungen infolge HIV-Krankheit [Humane Immundefizienz-Viruskrankheit] AIDS/HIV icd B21

Sonstige näher bezeichnete Krankheiten infolge HIV-Krankheit [Humane Immundefizienz-Viruskrankheit] AIDS/HIV icd B22

Nicht näher bezeichnete HIV-Krankheit [Humane Immundefizienz-Viruskrankheit] AIDS/HIV icd B24

Hodgkin-Lymphom [Lymphogranulomatose] Lymphoma icd C81

Follikuläres Lymphom Lymphoma icd C82

Nicht follikuläres Lymphom Lymphoma icd C83

Reifzellige T/NK-Zell-Lymphome Lymphoma icd C84

Sonstige und nicht näher bezeichnete Typen des Non-Hodgkin-Lymphoms Lymphoma icd C85

Bösartige immunproliferative Krankheiten Lymphoma icd C88

Sonstige und nicht näher bezeichnete bösartige Neubildungen des lymphatischen, blutbildenden und verwandten Gewebes Lymphoma icd C96

Multiples Myelom Lymphoma icd C90.0

Extramedulläres Plasmozytom Lymphoma icd C90.2

Sekundäre und nicht näher bezeichnete bösartige Neubildung der Lymphknoten Metastatic cancer icd C77

Sekundäre bösartige Neubildung der Atmungs- und Verdauungsorgane Metastatic cancer icd C78

Sekundäre bösartige Neubildung an sonstigen und nicht näher bezeichneten Lokalisationen Metastatic cancer icd C79

Bösartige Neubildung ohne Angabe der Lokalisation Metastatic cancer icd C80

Bösartige Neubildung der Lippe Solid tumor without metastasis icd C00

Bösartige Neubildung des Zungengrundes Solid tumor without metastasis icd C01

Bösartige Neubildung sonstiger und nicht näher bezeichneter Teile der Zunge Solid tumor without metastasis icd C02

Bösartige Neubildung des Zahnfleisches Solid tumor without metastasis icd C03

Bösartige Neubildung des Mundbodens Solid tumor without metastasis icd C04

Bösartige Neubildung des Gaumens Solid tumor without metastasis icd C05

Bösartige Neubildung sonstiger und nicht näher bezeichneter Teile des Mundes Solid tumor without metastasis icd C06

Bösartige Neubildung der Parotis Solid tumor without metastasis icd C07

Bösartige Neubildung sonstiger und nicht näher bezeichneter großer Speicheldrüsen Solid tumor without metastasis icd C08

Bösartige Neubildung der Tonsille Solid tumor without metastasis icd C09

Bösartige Neubildung des Oropharynx Solid tumor without metastasis icd C10

Bösartige Neubildung des Nasopharynx Solid tumor without metastasis icd C11

Bösartige Neubildung des Recessus piriformis Solid tumor without metastasis icd C12

Bösartige Neubildung des Hypopharynx Solid tumor without metastasis icd C13

Bösartige Neubildung sonstiger und ungenau bezeichneter Lokalisationen der Lippe, der Mundhöhle und des Pharynx Solid tumor without metastasis icd C14

Bösartige Neubildung des Ösophagus Solid tumor without metastasis icd C15

Bösartige Neubildung des Magens Solid tumor without metastasis icd C16

Bösartige Neubildung des Dünndarmes Solid tumor without metastasis icd C17

Bösartige Neubildung des Kolons Solid tumor without metastasis icd C18

Bösartige Neubildung am Rektosigmoid, Übergang Solid tumor without metastasis icd C19

Bösartige Neubildung des Rektums Solid tumor without metastasis icd C20

Bösartige Neubildung des Anus und des Analkanals Solid tumor without metastasis icd C21

Bösartige Neubildung der Leber und der intrahepatischen Gallengänge Solid tumor without metastasis icd C22

Bösartige Neubildung der Gallenblase Solid tumor without metastasis icd C23

Bösartige Neubildung sonstiger und nicht näher bezeichneter Teile der Gallenwege Solid tumor without metastasis icd C24

Bösartige Neubildung des Pankreas Solid tumor without metastasis icd C25

Bösartige Neubildung sonstiger und ungenau bezeichneter Verdauungsorgane Solid tumor without metastasis icd C26

Bösartige Neubildung der Nasenhöhle und des Mittelohres Solid tumor without metastasis icd C30

Bösartige Neubildung der Nasennebenhöhlen Solid tumor without metastasis icd C31

Bösartige Neubildung des Larynx Solid tumor without metastasis icd C32

Bösartige Neubildung der Trachea Solid tumor without metastasis icd C33

Bösartige Neubildung der Bronchien und der Lunge Solid tumor without metastasis icd C34

Bösartige Neubildung des Thymus Solid tumor without metastasis icd C37

Bösartige Neubildung des Herzens, des Mediastinums und der Pleura Solid tumor without metastasis icd C38

Bösartige Neubildung sonstiger und ungenau bezeichneter Lokalisationen des Atmungssystems und sonstiger intrathorakaler Organe Solid tumor without metastasis icd C39

Bösartige Neubildung des Knochens und des Gelenkknorpels der Extremitäten Solid tumor without metastasis icd C40

| Bösartige Neubildung des Knochens und des Gelenkknorpels sonstiger und nicht näher bezeichneter Lokalisationen | Solid tumor without metastasis icd | C41 |
| --- | --- | --- |
| Bösartiges Melanom der Haut | Solid tumor without metastasis icd | C43 |
| Mesotheliom | Solid tumor without metastasis icd | C45 |
| Kaposi-Sarkom [Sarcoma idiopathicum multiplex haemorrhagicum] | Solid tumor without metastasis icd | C46 |
| Bösartige Neubildung der peripheren Nerven und des autonomen Nervensystems | Solid tumor without metastasis icd | C47 |
| Bösartige Neubildung des Retroperitoneums und des Peritoneums | Solid tumor without metastasis icd | C48 |
| Bösartige Neubildung sonstigen Bindegewebes und anderer Weichteilgewebe | Solid tumor without metastasis icd | C49 |
| Bösartige Neubildung der Brustdrüse [Mamma] | Solid tumor without metastasis icd | C50 |
| Bösartige Neubildung der Vulva | Solid tumor without metastasis icd | C51 |
| Bösartige Neubildung der Vagina | Solid tumor without metastasis icd | C52 |
| Bösartige Neubildung der Cervix uteri | Solid tumor without metastasis icd | C53 |
| Bösartige Neubildung des Corpus uteri | Solid tumor without metastasis icd | C54 |
| Bösartige Neubildung des Uterus, Teil nicht näher bezeichnet | Solid tumor without metastasis icd | C55 |
| Bösartige Neubildung des Ovars | Solid tumor without metastasis icd | C56 |
| Bösartige Neubildung sonstiger und nicht näher bezeichneter weiblicher Genitalorgane | Solid tumor without metastasis icd | C57 |
| Bösartige Neubildung der Plazenta | Solid tumor without metastasis icd | C58 |
| Bösartige Neubildung des Penis | Solid tumor without metastasis icd | C60 |
| Bösartige Neubildung der Prostata | Solid tumor without metastasis icd | C61 |
| Bösartige Neubildung des Hodens | Solid tumor without metastasis icd | C62 |
| Bösartige Neubildung sonstiger und nicht näher bezeichneter männlicher Genitalorgane | Solid tumor without metastasis icd | C63 |
| Bösartige Neubildung der Niere, ausgenommen Nierenbecken | Solid tumor without metastasis icd | C64 |
| Bösartige Neubildung des Nierenbeckens | Solid tumor without metastasis icd | C65 |
| Bösartige Neubildung des Ureters | Solid tumor without metastasis icd | C66 |
| Bösartige Neubildung der Harnblase | Solid tumor without metastasis icd | C67 |
| Bösartige Neubildung sonstiger und nicht näher bezeichneter Harnorgane | Solid tumor without metastasis icd | C68 |
| Bösartige Neubildung des Auges und der Augenanhangsgebilde | Solid tumor without metastasis icd | C69 |
| Bösartige Neubildung der Meningen | Solid tumor without metastasis icd | C70 |
| Bösartige Neubildung des Gehirns | Solid tumor without metastasis icd | C71 |
| Bösartige Neubildung des Rückenmarkes, der Hirnnerven und anderer Teile des Zentralnervensystems | Solid tumor without metastasis icd | C72 |
| Bösartige Neubildung der Schilddrüse | Solid tumor without metastasis icd | C73 |
| Bösartige Neubildung der Nebenniere | Solid tumor without metastasis icd | C74 |
| Bösartige Neubildung sonstiger endokriner Drüsen und verwandter Strukturen | Solid tumor without metastasis icd | C75 |
| Bösartige Neubildung sonstiger und ungenau bezeichneter Lokalisationen | Solid tumor without metastasis icd | C76 |
| Bösartige Neubildungen als Primärtumoren an mehreren Lokalisationen | Solid tumor without metastasis icd | C97 |
| Sclerodermia circumscripta [Morphaea] | Rheumatoid arthritis/ collagen v icd | L94.0 |
| Lineare oder bandförmige Sklerodermie | Rheumatoid arthritis/ collagen v icd | L94.1 |
| Sklerodaktylie | Rheumatoid arthritis/ collagen v icd | L94.3 |
| Seropositive chronische Polyarthritis | Rheumatoid arthritis/ collagen v icd | M05 |
| Sonstige chronische Polyarthritis | Rheumatoid arthritis/ collagen v icd | M06 |
| Juvenile Arthritis | Rheumatoid arthritis/ collagen v icd | M08 |
| Chronische postrheumatische Arthritis [Jaccoud-Arthritis] | Rheumatoid arthritis/ collagen v icd | M12.0 |
| Palindromer Rheumatismus | Rheumatoid arthritis/ collagen v icd | M12.3 |
| Panarteriitis nodosa und verwandte Zustände | Rheumatoid arthritis/ collagen v icd | M30 |
| Hypersensitivitätsangiitis | Rheumatoid arthritis/ collagen v icd | M31.0 |
| Thrombotische Mikroangiopathie | Rheumatoid arthritis/ collagen v icd | M31.1 |
| Thrombotische Mikroangiopathie | Rheumatoid arthritis/ collagen v icd | M31.2 |
| Wegener-Granulomatose | Rheumatoid arthritis/ collagen v icd | M31.3 |
| Systemischer Lupus erythematodes | Rheumatoid arthritis/ collagen v icd | M32 |
| Dermatomyositis-Polymyositis | Rheumatoid arthritis/ collagen v icd | M33 |
| Systemische Sklerose | Rheumatoid arthritis/ collagen v icd | M34 |
| Sonstige Krankheiten mit Systembeteiligung des Bindegewebes | Rheumatoid arthritis/ collagen v icd | M35 |
| Spondylitis ankylosans | Rheumatoid arthritis/ collagen v icd | M45 |
| Sakroiliitis, anderenorts nicht klassifiziert | Rheumatoid arthritis/ collagen v icd | M46.1 |
| Sonstige näher bezeichnete entzündliche Spondylopathien | Rheumatoid arthritis/ collagen v icd | M46.8 |
| Entzündliche Spondylopathie, nicht näher bezeichnet | Rheumatoid arthritis/ collagen v icd | M46.9 |
| Thalassämie | Coagulaopathy icd | D56 |
| Sichelzellenkrankheiten | Coagulaopathy icd | D57 |
| Sonstige hereditäre hämolytische Anämien | Coagulaopathy icd | D58 |
| Erworbene hämolytische Anämien | Coagulaopathy icd | D59 |
| Erworbene isolierte aplastische Anämie [Erythroblastopenie] [pure red cell aplasia] | Coagulaopathy icd | D60 |
| Sonstige aplastische Anämien | Coagulaopathy icd | D61 |
| Akute Blutungsanämie | Coagulaopathy icd | D62 |
| Anämie bei chronischen, anderenorts klassifizierten Krankheiten | Coagulaopathy icd | D63 |
| Sonstige Anämien | Coagulaopathy icd | D64 |
| Disseminierte intravasale Gerinnung [Defibrinationssyndrom] | Coagulaopathy icd | D65 |
| Hereditärer Faktor-VIII-Mangel | Coagulaopathy icd | D66 |
| Hereditärer Faktor-IX-Mangel | Coagulaopathy icd | D67 |
| Sonstige Koagulopathien | Coagulaopathy icd | D68 |
| Purpura und sonstige hämorrhagische Diathesen | Coagulaopathy icd | D69.1 |
| Purpura und sonstige hämorrhagische Diathesen | Coagulaopathy icd | D69.3 |
| Purpura und sonstige hämorrhagische Diathesen | Coagulaopathy icd | D69.4 |
| Purpura und sonstige hämorrhagische Diathesen | Coagulaopathy icd | D69.5 |
| Purpura und sonstige hämorrhagische Diathesen | Coagulaopathy icd | D69.6 |
| Adipositas | Obesity icd | E66 |
| Kwashiorkor | Weight loss icd | E40 |
| Alimentärer Marasmus | Weight loss icd | E41 |
| Kwashiorkor-Marasmus | Weight loss icd | E42 |
| Nicht näher bezeichnete erhebliche Energie- und Eiweißmangelernährung | Weight loss icd | E43 |
| Energie- und Eiweißmangelernährung mäßigen und leichten Grades | Weight loss icd | E44 |
| Entwicklungsverzögerung durch Energie- und Eiweißmangelernährung | Weight loss icd | E45 |
| Nicht näher bezeichnete Energie- und Eiweißmangelernährung | Weight loss icd | E46 |
| Abnorme Gewichtsabnahme | Weight loss icd | R63.4 |
| Kachexie | Weight loss icd | R64 |
| Syndrom der inadäquaten Sekretion von Adiuretin | Fluid and electrolyte disorders icd | E22.2 |
| Volumenmangel | Fluid and electrolyte disorders icd | E86 |
| Sonstige Störungen des Wasser- und Elektrolythaushaltes sowie des Säure-Basen-Gleichgewichts | Fluid and electrolyte disorders icd | E87 |
| Eisenmangelanämie nach Blutverlust (chronisch) | Blood loss anaemia icd | D50.0 |
| Sonstige Eisenmangelanämien | Deficiency anemia icd | D50.8 |
| Eisenmangelanämie, nicht näher bezeichnet | Deficiency anemia icd | D50.9 |
| Vitamin-B12-Mangelanämie | Deficiency anemia icd | D51 |
| Folsäure-Mangelanämie | Deficiency anemia icd | D52 |
| Sonstige alimentäre Anämien | Deficiency anemia icd | D53 |
| Psychische und Verhaltensstörungen durch Alkohol | Alcohol abuse icd | F10 |
| Niazinmangel [Pellagra] | Alcohol abuse icd | E52 |
| Alkohol-Polyneuropathie | Alcohol abuse icd | G62.1 |
| Alkoholische Kardiomyopathie | Alcohol abuse icd | I42.6 |
| Alkoholgastritis | Alcohol abuse icd | K29.2 |
| Alkoholische Leberkrankheit | Alcohol abuse icd | K70 |
| Alkoholische Leberzirrhose | Alcohol abuse icd | K70.3 |
| Alkoholische Leberkrankheit, nicht näher bezeichnet | Alcohol abuse icd | K70.9 |
| Toxische Wirkung von Alkohol | Alcohol abuse icd | T51 |
| Rehabilitationsmaßnahmen bei Alkoholismus | Alcohol abuse icd | Z50.2 |
| #NV | Alcohol abuse icd | Z71.4 |
| #NV | Alcohol abuse icd | Z72.1 |
| Psychische und Verhaltensstörungen durch Opioide | Drug abuse icd | F11 |
| Psychische und Verhaltensstörungen durch Cannabinoide | Drug abuse icd | F12 |
| Psychische und Verhaltensstörungen durch Sedativa oder Hypnotika | Drug abuse icd | F13 |
| Psychische und Verhaltensstörungen durch Kokain | Drug abuse icd | F14 |
| Psychische und Verhaltensstörungen durch andere Stimulanzien, einschließlich Koffein | Drug abuse icd | F15 |
| Psychische und Verhaltensstörungen durch Halluzinogene | Drug abuse icd | F16 |
| Psychische und Verhaltensstörungen durch flüchtige Lösungsmittel | Drug abuse icd | F18 |
| Psychische und Verhaltensstörungen durch multiplen Substanzgebrauch und Konsum anderer psychotroper Substanzen | Drug abuse icd | F19 |
| Nicht belegte Schlüsselnummer U99.9! | Drug abuse icd | Z71.5 |
| Nicht belegte Schlüsselnummer U99.9! | Drug abuse icd | Z72.2 |
| Schizophrenie | Psychosis icd | F20 |
| Anhaltende wahnhafte Störungen | Psychosis icd | F22 |
| Akute vorübergehende psychotische Störungen | Psychosis icd | F23 |
| Induzierte wahnhafte Störung | Psychosis icd | F24 |
| Schizoaffektive Störungen | Psychosis icd | F25 |
| Sonstige nichtorganische psychotische Störungen | Psychosis icd | F28 |
| Nicht näher bezeichnete nichtorganische Psychose | Psychosis icd | F29 |
| Manie mit psychotischen Symptomen | Psychosis icd | F30.2 |
| Bipolare affektive Störung, gegenwärtig manische Episode mit psychotischen Symptomen | Psychosis icd | F31.2 |
| Bipolare affektive Störung, gegenwärtig schwere depressive Episode mit psychotischen Symptomen | Psychosis icd | F31.5 |
| Postschizophrene Depression | Depression icd | F20.4 |
| Bipolare affektive Störung, gegenwärtig leichte oder mittelgradige depressive Episode | Depression icd | F31.3 |
| Bipolare affektive Störung, gegenwärtig schwere depressive Episode ohne psychotische Symptome | Depression icd | F31.4 |
| Bipolare affektive Störung, gegenwärtig schwere depressive Episode mit psychotischen Symptomen | Depression icd | F31.5 |
| Depressive Episode | Depression icd | F32 |
| Rezidivierende depressive Störung | Depression icd | F33 |
| Dysthymia | Depression icd | F34.1 |
| Angst und depressive Störung, gemischt | Depression icd | F41.2 |
| Anpassungsstörungen | Depression icd | F43.2 |
| Rheumatische Herzkrankheit, nicht näher bezeichnet | Congestive heart failure icd | I09.9 |
| Hypertensive Herzkrankheit mit (kongestiver) Herzinsuffizienz | Congestive heart failure icd | I11.0 |
| Hypertensive Herz- und Nierenkrankheit mit (kongestiver) Herzinsuffizienz | Congestive heart failure icd | I13.0 |
| Hypertensive Herz- und Nierenkrankheit mit (kongestiver) Herzinsuffizienz und Niereninsuffizienz | Congestive heart failure icd | I13.2 |
| Ischämische Kardiomyopathie | Congestive heart failure icd | I25.5 |
| Dilatative Kardiomyopathie | Congestive heart failure icd | I42.0 |
| Sonstige restriktive Kardiomyopathie | Congestive heart failure icd | I42.5 |
| Alkoholische Kardiomyopathie | Congestive heart failure icd | I42.6 |
| Kardiomyopathie durch Arzneimittel oder sonstige exogene Substanzen | Congestive heart failure icd | I42.7 |
| Sonstige Kardiomyopathien | Congestive heart failure icd | I42.8 |
| Kardiomyopathie, nicht näher bezeichnet | Congestive heart failure icd | I42.9 |
| Kardiomyopathie bei anderenorts klassifizierten Krankheiten | Congestive heart failure icd | I43 |
| Herzinsuffizienz | Congestive heart failure icd | I50 |
| Herzinsuffizienz beim Neugeborenen | Congestive heart failure icd | P29.0 |
| Atrioventrikulärer Block 2. Grades | Cardiac arrhythmias icd | I44.1 |
| Atrioventrikulärer Block 3. Grades | Cardiac arrhythmias icd | I44.2 |
| Sonstiger und nicht näher bezeichneter atrioventrikulärer Block | Cardiac arrhythmias icd | I44.3 |
| Präexzitations-Syndrom | Cardiac arrhythmias icd | I45.6 |
| Kardiale Erregungsleitungsstörung, nicht näher bezeichnet | Cardiac arrhythmias icd | I45.9 |
| Paroxysmale Tachykardie | Cardiac arrhythmias icd | I47 |
| Vorhofflimmern und Vorhofflattern | Cardiac arrhythmias icd | I48 |
| Sonstige kardiale Arrhythmien | Cardiac arrhythmias icd | I49 |
| Tachykardie, nicht näher bezeichnet | Cardiac arrhythmias icd | R00.0 |
| Bradykardie, nicht näher bezeichnet | Cardiac arrhythmias icd | R00.1 |
| Sonstige und nicht näher bezeichnete Störungen des Herzschlages | Cardiac arrhythmias icd | R00.8 |
| Mechanische Komplikation durch ein kardiales elektronisches Gerät | Cardiac arrhythmias icd | T82.1 |
| Kardiovaskuläre Syphilis | Valvular disease icd | A52.0 |
| Rheumatische Mitralklappenkrankheiten | Valvular disease icd | I05 |
| Rheumatische Aortenklappenkrankheiten | Valvular disease icd | I06 |
| Rheumatische Trikuspidalklappenkrankheiten | Valvular disease icd | I07 |
| Krankheiten mehrerer Herzklappen | Valvular disease icd | I08 |
| Rheumatische Krankheiten des Endokards, Herzklappe nicht näher bezeichnet | Valvular disease icd | I09.1 |
| Sonstige näher bezeichnete rheumatische Herzkrankheiten | Valvular disease icd | I09.8 |
| Nichtrheumatische Mitralklappenkrankheiten | Valvular disease icd | I34 |
| Nichtrheumatische Aortenklappenkrankheiten | Valvular disease icd | I35 |
| Nichtrheumatische Trikuspidalklappenkrankheiten | Valvular disease icd | I36 |
| Pulmonalklappenkrankheiten | Valvular disease icd | I37 |
| Endokarditis, Herzklappe nicht näher bezeichnet | Valvular disease icd | I38 |
| Endokarditis und Herzklappenkrankheiten bei anderenorts klassifizierten Krankheiten | Valvular disease icd | I39 |
| Angeborene Aortenklappenstenose | Valvular disease icd | Ǫ23.0 |

| Angeborene Aortenklappeninsuffizienz | Valvular disease | icd | Ǫ23.1 |
| --- | --- | --- | --- |
| Angeborene Mitralklappenstenose | Valvular disease | icd | Ǫ23.2 |
| Angeborene Mitralklappeninsuffizienz | Valvular disease | icd | Ǫ23.3 |
| Vorhandensein einer künstlichen Herzklappe | Valvular disease | icd | Z95.2 |
| Vorhandensein eines anderen Herzklappenersatzes | Valvular disease | icd | Z95.4 |
| Lungenembolie | Pulmonary circulation disorders | icd | I26 |
| Sonstige pulmonale Herzkrankheiten | Pulmonary circulation disorders | icd | I27 |
| Arteriovenöse Fistel der Lungengefäße | Pulmonary circulation disorders | icd | I28.0 |
| Sonstige näher bezeichnete Krankheiten der Lungengefäße | Pulmonary circulation disorders | icd | I28.8 |
| Krankheit der Lungengefäße, nicht näher bezeichnet | Pulmonary circulation disorders | icd | I28.9 |
| Atherosklerose | Peripheral vascular disorders | icd | I70 |
| Aortenaneurysma und -dissektion | Peripheral vascular disorders | icd | I71 |
| Thrombangiitis obliterans [Endangiitis von-Winiwarter-Buerger] | Peripheral vascular disorders | icd | I73.1 |
| Sonstige näher bezeichnete periphere Gefäßkrankheiten | Peripheral vascular disorders | icd | I73.8 |
| Periphere Gefäßkrankheit, nicht näher bezeichnet | Peripheral vascular disorders | icd | I73.9 |
| Arterienstriktur | Peripheral vascular disorders | icd | I77.1 |
| Aortenaneurysma bei anderenorts klassifizierten Krankheiten | Peripheral vascular disorders | icd | I79.0 |
| Periphere Angiopathie bei anderenorts klassifizierten Krankheiten | Peripheral vascular disorders | icd | I79.2 |
| Chronische Gefäßkrankheiten des Darmes | Peripheral vascular disorders | icd | K55.1 |
| Sonstige Gefäßkrankheiten des Darmes | Peripheral vascular disorders | icd | K55.8 |
| Gefäßkrankheit des Darmes, nicht näher bezeichnet | Peripheral vascular disorders | icd | K55.9 |
| Nicht belegte Schlüsselnummer U99.9! | Peripheral vascular disorders | icd | Z95.8 |
| Nicht belegte Schlüsselnummer U99.9! | Peripheral vascular disorders | icd | Z95.9 |
| Essentielle (primäre) Hypertonie | Hypertension uncomlicated | icd | I10 |
| Hypertensive Herzkrankheit | Hypertension complicated | icd | I11 |
| Hypertensive Nierenkrankheit | Hypertension complicated | icd | I12 |
| Hypertensive Herz- und Nierenkrankheit | Hypertension complicated | icd | I13 |
| Sekundäre Hypertonie | Hypertension complicated | icd | I15 |
| Humane T-Zell-lymphotrope Virus-assoziierte Myelopathie | Paralysis | icd | G04.1 |
| Hereditäre spastische Paraplegie | Paralysis | icd | G11.4 |
| Spastische diplegische Zerebralparese | Paralysis | icd | G80.1 |
| Infantile hemiplegische Zerebralparese | Paralysis | icd | G80.2 |
| Hemiparese und Hemiplegie | Paralysis | icd | G81 |
| Paraparese und Paraplegie, Tetraparese und Tetraplegie | Paralysis | icd | G82 |
| Diparese und Diplegie der oberen Extremitäten | Paralysis | icd | G83.0 |
| Monoparese und Monoplegie einer unteren Extremität | Paralysis | icd | G83.1 |
| Monoparese und Monoplegie einer oberen Extremität | Paralysis | icd | G83.2 |
| Monoparese und Monoplegie, nicht näher bezeichnet | Paralysis | icd | G83.3 |
| Cauda- (equina-) Syndrom | Paralysis | icd | G83.4 |
| Lähmungssyndrom, nicht näher bezeichnet | Paralysis | icd | G83.9 |
| Chorea Huntington | Other neurolog disorders | icd | G10 |
| Systematrophien, vorwiegend das Zentralnervensystem betreffend, bei anderenorts klassifizierten Krankheiten | Other neurolog disorders | icd | G13 |
| Primäres Parkinson-Syndrom | Other neurolog disorders | icd | G20 |
| Parkinson-Syndrom bei anderenorts klassifizierten Krankheiten | Other neurolog disorders | icd | G22 |
| Arzneimittelinduzierte Chorea | Other neurolog disorders | icd | G25.4 |
| Sonstige Chorea | Other neurolog disorders | icd | G25.5 |
| Degeneration des Nervensystems durch Alkohol | Other neurolog disorders | icd | G31.2 |
| Sonstige näher bezeichnete degenerative Krankheiten des Nervensystems | Other neurolog disorders | icd | G31.8 |
| Degenerative Krankheit des Nervensystems, nicht näher bezeichnet | Other neurolog disorders | icd | G31.9 |
| Sonstige degenerative Krankheiten des Nervensystems bei anderenorts klassifizierten Krankheiten | Other neurolog disorders | icd | G32 |
| Multiple Sklerose [Encephalomyelitis disseminata] | Other neurolog disorders | icd | G35 |
| Sonstige akute disseminierte Demyelinisation | Other neurolog disorders | icd | G36 |
| Sonstige demyelinisierende Krankheiten des Zentralnervensystems | Other neurolog disorders | icd | G37 |
| Epilepsie | Other neurolog disorders | icd | G40 |
| Status epilepticus | Other neurolog disorders | icd | G41 |
| Anoxische Hirnschädigung, anderenorts nicht klassifiziert | Other neurolog disorders | icd | G93.1 |
| Enzephalopathie, nicht näher bezeichnet | Other neurolog disorders | icd | G93.4 |
| Dysphasie und Aphasie | Other neurolog disorders | icd | R47.0 |
| Krämpfe, anderenorts nicht klassifiziert | Other neurolog disorders | icd | R56 |
| Aneurysma und Dissektion sonstiger näher bezeichneter Arterien | chronic pulmonary disease | icd | I72.8 |
| Aneurysma und Dissektion nicht näher bezeichneter Lokalisation | chronic pulmonary disease | icd | I72.9 |
| Bronchitis, nicht als akut oder chronisch bezeichnet | chronic pulmonary disease | icd | J40 |
| Einfache und schleimig-eitrige chronische Bronchitis | chronic pulmonary disease | icd | J41 |
| Nicht näher bezeichnete chronische Bronchitis | chronic pulmonary disease | icd | J42 |
| Emphysem | chronic pulmonary disease | icd | J43 |
| Sonstige chronische obstruktive Lungenkrankheit | chronic pulmonary disease | icd | J44 |
| Asthma bronchiale | chronic pulmonary disease | icd | J45 |
| Status asthmaticus | chronic pulmonary disease | icd | J46 |
| Bronchiektasen | chronic pulmonary disease | icd | J47 |
| Kohlenbergarbeiter-Pneumokoniose | chronic pulmonary disease | icd | J60 |
| Pneumokoniose durch Asbest und sonstige anorganische Fasern | chronic pulmonary disease | icd | J61 |
| Pneumokoniose durch Ǫuarzstaub | chronic pulmonary disease | icd | J62 |
| Pneumokoniose durch sonstige anorganische Stäube | chronic pulmonary disease | icd | J63 |
| Nicht näher bezeichnete Pneumokoniose | chronic pulmonary disease | icd | J64 |
| Pneumokoniose in Verbindung mit Tuberkulose | chronic pulmonary disease | icd | J65 |
| Krankheit der Atemwege durch spezifischen organischen Staub | chronic pulmonary disease | icd | J66 |
| Allergische Alveolitis durch organischen Staub | chronic pulmonary disease | icd | J67 |
| Chronische Krankheiten der Atmungsorgane durch chemische Substanzen, Gase, Rauch und Dämpfe | chronic pulmonary disease | icd | J68.4 |
| Chronische und sonstige Lungenbeteiligung bei Strahleneinwirkung | chronic pulmonary disease | icd | J70.1 |
| Chronische arzneimittelinduzierte interstitielle Lungenkrankheiten | chronic pulmonary disease | icd | J70.3 |
| Diabetes mellitus, Typ 1: Mit Koma | Diabtes uncomplicated | icd | E10.0 |
| Diabetes mellitus, Typ 1: Mit Ketoazidose | Diabtes uncomplicated | icd | E10.1 |
| Diabetes mellitus, Typ 1: Ohne Komplikationen | Diabtes uncomplicated | icd | E10.9 |
| Diabetes mellitus, Typ 2: Mit Koma | Diabtes uncomplicated | icd | E11.0 |
| Diabetes mellitus, Typ 2: Mit Ketoazidose | Diabtes uncomplicated | icd | E11.1 |
| Diabetes mellitus, Typ 2: Ohne Komplikationen | Diabtes uncomplicated | icd | E11.9 |
| Diabetes mellitus in Verbindung mit Fehl- oder Mangelernährung [Malnutrition]: Mit Koma | Diabtes uncomplicated | icd | E12.0 |
| Diabetes mellitus in Verbindung mit Fehl- oder Mangelernährung [Malnutrition]: Mit Ketoazidose | Diabtes uncomplicated | icd | E12.1 |
| Diabetes mellitus in Verbindung mit Fehl- oder Mangelernährung [Malnutrition]: Ohne Komplikationen | Diabtes uncomplicated | icd | E12.9 |
| Sonstiger näher bezeichneter Diabetes mellitus: Mit Koma | Diabtes uncomplicated | icd | E13.0 |
| Sonstiger näher bezeichneter Diabetes mellitus: Mit Ketoazidose | Diabtes uncomplicated | icd | E13.1 |
| Sonstiger näher bezeichneter Diabetes mellitus: Ohne Komplikationen | Diabtes uncomplicated | icd | E13.9 |
| Nicht näher bezeichneter Diabetes mellitus: Mit Koma | Diabtes uncomplicated | icd | E14.0 |
| Nicht näher bezeichneter Diabetes mellitus: Mit Ketoazidose | Diabtes uncomplicated | icd | E14.1 |
| Nicht näher bezeichneter Diabetes mellitus: Ohne Komplikationen | Diabtes uncomplicated | icd | E14.9 |
| Diabetes mellitus, Typ 1: Mit Nierenkomplikationen | Diabtes complicated | icd | E10.2 |
| Diabetes mellitus, Typ 1: Mit Augenkomplikationen | Diabtes complicated | icd | E10.3 |
| Diabetes mellitus, Typ 1: Mit neurologischen Komplikationen | Diabtes complicated | icd | E10.4 |
| Diabetes mellitus, Typ 1: Mit peripheren vaskulären Komplikationen | Diabtes complicated | icd | E10.5 |
| Diabetes mellitus, Typ 1: Mit sonstigen näher bezeichneten Komplikationen | Diabtes complicated | icd | E10.6 |
| Diabetes mellitus, Typ 1: Mit multiplen Komplikationen | Diabtes complicated | icd | E10.7 |
| Diabetes mellitus, Typ 1: Mit nicht näher bezeichneten Komplikationen | Diabtes complicated | icd | E10.8 |
| Diabetes mellitus, Typ 2: Mit Nierenkomplikationen | Diabtes complicated | icd | E11.2 |
| Diabetes mellitus, Typ 2: Mit Augenkomplikationen | Diabtes complicated | icd | E11.3 |
| Diabetes mellitus, Typ 2: Mit neurologischen Komplikationen | Diabtes complicated | icd | E11.4 |
| Diabetes mellitus, Typ 2: Mit peripheren vaskulären Komplikationen | Diabtes complicated | icd | E11.5 |
| Diabetes mellitus, Typ 2: Mit sonstigen näher bezeichneten Komplikationen | Diabtes complicated | icd | E11.6 |
| Diabetes mellitus, Typ 2: Mit multiplen Komplikationen | Diabtes complicated | icd | E11.7 |
| Diabetes mellitus, Typ 2: Mit nicht näher bezeichneten Komplikationen | Diabtes complicated | icd | E11.8 |
| Diabetes mellitus in Verbindung mit Fehl- oder Mangelernährung [Malnutrition]: Mit Nierenkomplikationen | Diabtes complicated | icd | E12.2 |
| Diabetes mellitus in Verbindung mit Fehl- oder Mangelernährung [Malnutrition]: Mit Augenkomplikationen | Diabtes complicated | icd | E12.3 |
| Diabetes mellitus in Verbindung mit Fehl- oder Mangelernährung [Malnutrition]: Mit neurologischen Komplikationen | Diabtes complicated | icd | E12.4 |
| Diabetes mellitus in Verbindung mit Fehl- oder Mangelernährung [Malnutrition]: Mit peripheren vaskulären Komplikationen | Diabtes complicated | icd | E12.5 |
| Diabetes mellitus in Verbindung mit Fehl- oder Mangelernährung [Malnutrition]: Mit sonstigen näher bezeichneten Komplikationen | Diabtes complicated | icd | E12.6 |
| Diabetes mellitus in Verbindung mit Fehl- oder Mangelernährung [Malnutrition]: Mit multiplen Komplikationen | Diabtes complicated | icd | E12.7 |
| Diabetes mellitus in Verbindung mit Fehl- oder Mangelernährung [Malnutrition]: Mit nicht näher bezeichneten Komplikationen | Diabtes complicated | icd | E12.8 |
| Sonstiger näher bezeichneter Diabetes mellitus: Mit Nierenkomplikationen | Diabtes complicated | icd | E13.2 |
| Sonstiger näher bezeichneter Diabetes mellitus: Mit Augenkomplikationen | Diabtes complicated | icd | E13.3 |
| Sonstiger näher bezeichneter Diabetes mellitus: Mit neurologischen Komplikationen | Diabtes complicated | icd | E13.4 |
| Sonstiger näher bezeichneter Diabetes mellitus: Mit peripheren vaskulären Komplikationen | Diabtes complicated | icd | E13.5 |
| Sonstiger näher bezeichneter Diabetes mellitus: Mit sonstigen näher bezeichneten Komplikationen | Diabtes complicated | icd | E13.6 |
| Sonstiger näher bezeichneter Diabetes mellitus: Mit multiplen Komplikationen | Diabtes complicated | icd | E13.7 |
| Sonstiger näher bezeichneter Diabetes mellitus: Mit nicht näher bezeichneten Komplikationen | Diabtes complicated | icd | E13.8 |
| Nicht näher bezeichneter Diabetes mellitus: Mit Nierenkomplikationen | Diabtes complicated | icd | E14.2 |
| Nicht näher bezeichneter Diabetes mellitus: Mit Augenkomplikationen | Diabtes complicated | icd | E14.3 |
| Nicht näher bezeichneter Diabetes mellitus: Mit neurologischen Komplikationen | Diabtes complicated | icd | E14.4 |
| Nicht näher bezeichneter Diabetes mellitus: Mit peripheren vaskulären Komplikationen | Diabtes complicated | icd | E14.5 |
| Nicht näher bezeichneter Diabetes mellitus: Mit sonstigen näher bezeichneten Komplikationen | Diabtes complicated | icd | E14.6 |
| Nicht näher bezeichneter Diabetes mellitus: Mit multiplen Komplikationen | Diabtes complicated | icd | E14.7 |
| Nicht näher bezeichneter Diabetes mellitus: Mit nicht näher bezeichneten Komplikationen | Diabtes complicated | icd | E14.8 |
| Angeborenes Jodmangelsyndrom | Hypothyriodism | icd | E00 |
| Jodmangelbedingte Schilddrüsenkrankheiten und verwandte Zustände | Hypothyriodism | icd | E01 |
| Subklinische Jodmangel-Hypothyreose | Hypothyriodism | icd | E02 |
| Sonstige Hypothyreose | Hypothyriodism | icd | E03 |
| Hypothyreose nach medizinischen Maßnahmen | Hypothyriodism | icd | E89.0 |
| Hypertensive Nierenkrankheit mit Niereninsuffizienz | Renal failure | icd | I12.0 |
| Hypertensive Herz- und Nierenkrankheit mit Niereninsuffizienz | Renal failure | icd | I13.1 |
| Chronische Nierenkrankheit | Renal failure | icd | N18 |
| Nicht näher bezeichnete Niereninsuffizienz | Renal failure | icd | N19 |
| Renale Osteodystrophie | Renal failure | icd | N25.0 |
| Vorbereitung auf die Dialyse | Renal failure | icd | Z49.0 |
| Extrakorporale Dialyse | Renal failure | icd | Z49.1 |
| Sonstige Dialyse | Renal failure | icd | Z49.2 |
| Chronische Virushepatitis | Liver disease | icd | B18 |
| Sonstige und nicht näher bezeichnete Infektionskrankheiten | Liver disease | icd | BI85 |
| Magenvarizen | Liver disease | icd | I86.4 |
| Ösophagus- und Magenvarizen bei anderenorts klassifizierten Krankheiten, ohne Angabe einer Blutung | Liver disease | icd | I98.2 |
| Alkoholische Leberkrankheit | Liver disease | icd | K70 |
| Toxische Leberkrankheit mit Lebernekrose | Liver disease | icd | K71.1 |
| Toxische Leberkrankheit mit chronisch-persistierender Hepatitis | Liver disease | icd | K71.3 |
| Toxische Leberkrankheit mit chronischer lobulärer Hepatitis | Liver disease | icd | K71.4 |
| Toxische Leberkrankheit mit Fibrose und Zirrhose der Leber | Liver disease | icd | K71.7 |
| Leberversagen, anderenorts nicht klassifiziert | Liver disease | icd | K72 |
| Chronische Hepatitis, anderenorts nicht klassifiziert | Liver disease | icd | K73 |
| Fibrose und Zirrhose der Leber | Liver disease | icd | K74 |
| Fettleber [fettige Degeneration], anderenorts nicht klassifiziert | Liver disease | icd | K76.0 |
| Zentrale hämorrhagische Lebernekrose | Liver disease | icd | K76.2 |
| Leberkrankheit, nicht näher bezeichnet | Liver disease | icd | K76.9 |
| Zustand nach Lebertransplantation | Liver disease | icd | Z94.4 |
| Source:  Ǫuan H, Sundararajan V, Halfon P, et al. Coding algorithms for defining Comorbidities in ICD-9-CM and ICD-10 administrative data. Med Care. 2005 Nov; 43(11): 1130-9. |  |  |  |
